# Comparison of Less Invasive Stabilization System Plate and Retrograde Intramedullary Nail in the Fixation of Femoral Supracondylar Fractures in the Elderly: A Biomechanical Study

**DOI:** 10.1111/os.12449

**Published:** 2019-04-15

**Authors:** Yu‐ren Du, Jian‐xiong Ma, Shuo Wang, Lei Sun, Ying Wang, Bin Lu, Hao‐hao Bai, Yong‐cheng Hu, Xin‐long Ma

**Affiliations:** ^1^ Department of Orthopaedics Tianjin Medical University General Hospital Tianjin China; ^2^ Tianjin Institute of Orthopaedics Tianjin Hospital Tianjin China; ^3^ Department of Orthopaedics Tianjin Hospital Tianjin China; ^4^ Department of Orthopaedics the No. 464 Hospital of People's Liberation Army Tianjin China

**Keywords:** Biomechanical testing, Comminuted femoral supracondylar fracture, Less invasive stabilization system plate, Retrograde intramedullary nail

## Abstract

**Objective:**

To compare the biomechanical stabilities of less invasive stabilization system (LISS) plate and retrograde intramedullary nail (IMN) for the comminuted femoral supracondylar fracture fractures in the elderly.

**Methods:**

Sixteen pairs of embalmed cadaver femurs were obtained to simulate a comminuted supracondylar femur fracture (AO/OTA33‐A3) gap model. All left‐side specimens were fixed with LISS plate, and retrograde IMN were applied to the right‐side specimens. All specimens were tested in torsional, axial and cyclic load mode on an Instron testing machine.

**Results:**

The mean torsional stiffness for LISS plate group was 34.1% greater than retrograde IMN group (2.90 *vs*. 1.91 Nm/degree, *P* = 0.002), but the mean axial stiffness was greater for the retrograde IMN (199.16 vs. 303.93 N/mm, *P* < 0.001). The total deformation of LISS plate caused by cyclic axial loading was greater than retrograde IMN (4.17 *vs*. 3.57 mm, *P* = 0.014). Significantly less mean irreversible deformation was detected in LISS plate than in retrograde IMN (1.64 *vs*. 1.69 mm, *P* = 0.699). Failure loads of the constructs were significantly different between the two groups (LISS plate: 2941±128 N; retrograde IMN: 4022±176 N, *P* < 0.001).

**Conclusion:**

For comminuted femoral supracondylar fractures in the elderly, the tested instruments can both maintain sufficient biomechanical stabilities, but retrograde IMN is superior to LISS plate in deformation of fracture site.

Comminuted fractures of the distal femur are common but a challenge for orthopaedic surgeons. Comminuted femoral supracondylar fractures refer to a fracture within 9 cm of the distal femur and tend to occur in young men and older women. They are often caused by high‐energy trauma or result from osteoporosis with low‐energy injuries^1^, ^2^. They account for approximately 6% of all femoral fractures^3^. Most are unstable fractures and difficult to fix firmly. The incidences of malformation, non‐healing, infection, and limited knee joint motion are relatively high. Surgical instruments for distal femur fractures mainly include external fixation, intramedullary nail systems, and locking plate systems, which are suitable for intra‐articular fractures, displaced fractures, and open fractures, as well as for cases with vascular injury^4^. Reshaping the axial alignment of lower extremities, providing enough stiffness and stability, and preventing joint stiffness through early activities are the critical aspects of treating this type of fracture.

Two habitually applied methods in treating femoral supracondylar fractures are retrograde intramedullary nailing (IMN) and less invasive stabilization system (LISS) plate^5^. These treatments have been credited with providing the best functional outcomes in both extra and intraarticular fractures, surpassing the outcomes of dynamic condylar screwing (DCS) by Chander *et al.* 6. Through inserting multiple angle locking screws, the LISS plate not only keeps the fracture end stable but can also position the percutaneous screw at the distal end of the femur to avoid excessive peeling of the periosteum of the metaphysis and the diaphysis, and is superior to condylar plates^7^. This design can avoid shearing force between nails and the plate under axial force, so there is no need to offset the friction force as for other ordinary plates, and the locking plate does not directly contact the bone surface, thereby protecting the blood supply, which effectively reduces complication rates^8^. At the same time, the strength of the locking plate is related to the total contact area of all screws and bones, so the plate system will only fail if all the screws fail^9^. Retrograde IMN are well matched with the force line of the femur, and the AO/ASIF recommends using it to fix supracondylar fractures of the femur. Older patients typically have osteoporosis and low bone mass. Therefore, when the articular surface is intact, the LISS plate is relatively simple to operate outside the joint and it is easy to achieve angle stability and stronger pull‐out force. Intramedullary fixation with multiple interlocks facilitates the protection of blood supply and early mobilization.

A clinical randomized prospective study compared the outcomes for LISS plate *versus* retrograde IMN in the management of extra articular supracondylar femur fractures, and indicated that the complication rates were equivalent between them. The retrograde IMN achieved earlier union as well as better functional outcomes, and it was suggested that surgical planning and expertise rather than the choice of implant were crucial for optimal results^10^. LISS plate and retrograde IMN are universally adopted options for clinical treatment of comminuted femoral supracondylar fractures, and clinical results have demonstrated good clinical performance^11^, ^12^, ^13^, ^14^, ^15^.

However, there is no agreement on which internal fixation technique provides better stability through comparison of biomechanical properties^16^, ^17^, leading us to undertake this biomechanical comparative study. We constructed femoral supracondylar fracture models (AO/OTA33.A3) with LISS plates and retrograde IMN to: (i) evaluate the biomechanical properties in relation to axial and torsional stiffness, total deformation, and irreversible deformation of the two fixation techniques in the elderly; (ii) compare the stability of the two internal fixation techniques in treating femoral supracondylar fractures to further support clinical treatment; and (iii) define the failure load of the two internal fixation techniques in a femoral supracondylar fractures model to further assess the timing and mode of postoperative functional exercise.

## Materials and Methods

### *Specimens Selection and Pretreatment*

Sixteen pairs of embalmed intact adult cadaver femurs of elderly patients were obtained, with individuals’ ages ranging from 65 to 89 years (mean, 78.8 years). The specimens were from 11 males and 5 females, and were randomly assigned to an LISS plate group and a retrograde IMN group. All femurs were embalmed for 9–12 months. Attached soft tissues such as muscles and ligaments were stripped off before implantation and testing. We excluded bone tumors and other bone diseases which might affect the later experimental results using radiographs. All specimens were screened by dual energy X‐ray absorptiometry (DEXA, Hologic, Bedford, MA, USA) to determine whether there was osteoporosis at the distal femur.

This experiment was approved by the ethics committee of Tianjin Hospital.

### *Parameters of Internal Fixation Methods*

Titanium alloy LISS plates were applied to the left side of all cadaveric femoral specimens with five locking screws and four double cortical screws; all screws were tightened with a 4‐Nm torque limiter. Retrograde IMN were used for the femoral specimens of the right side, and two inward screws were implanted at the proximal and distal ends. Both LISS plates and retrograde IMN (donated by Da Bo Yingjing Medical Instrument Corporation, China) are widely used in clinical treatment in China.

### *Modeling Procedure*

**Figure 1:**
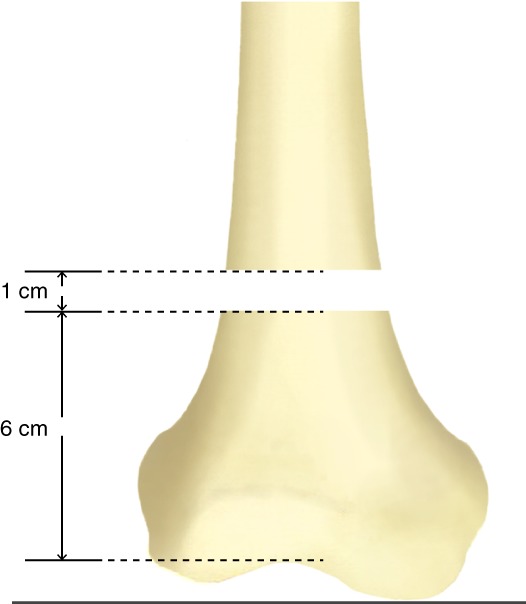
Illustration for AO/OTA33.A3 fracture gap model.

Transverse osteotomy of 1‐cm bone defect was created at a distance of 6 cm from the intercondylar fossa of the distal femur to simulate AO/OTA33.A3 comminuted femoral supracondylar unstable fractures (Fig. 1).

After completing the above steps, distal femur of approximately 30 cm was sawn off and reserved for the next tests. All fixations were performed by the same orthopedic surgeon in the same manner, thereby ensuring the correctness and firmness of the internal fixation and uniformity of the fracture model. The gap between the fracture fragments was 1 cm.

### *Radiological Examination before Tests*

**Figure 2:**
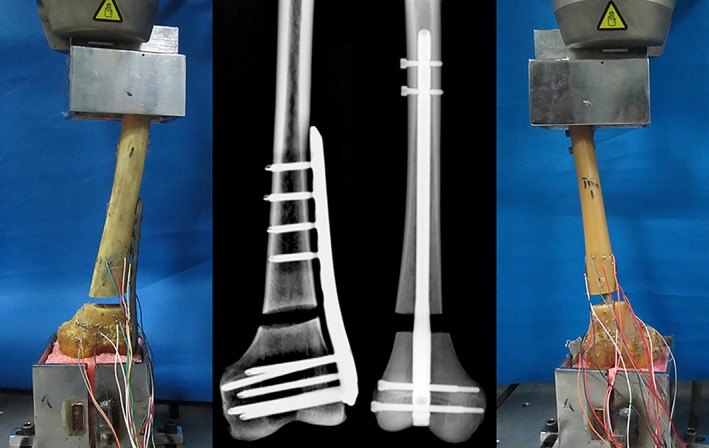
Radiographs of internal fixation of the distal femur and biomechanical loading experiment on Instron testing machine.

X‐ray examination of all specimens was undertaken after implantation surgery to ensure the optimal implant position was obtained. The X‐ray radiographs indicated the final position of the implants in the distal femur (Fig. 2).

### *Biomechanical Test*

Each specimen of distal femurs was embedded and fixed in a metal mold with polymethylmethacrylate (PMMA), ensuring no contact with PMMA and the implant. During the embedding process, it was ensured that the long axis of the femur was adducted 9° at the coronal plane to simulate a physiological single‐leg stance. The proximal femur was also embedded in a PMMA cup and placed in a mechanical load cell to reduce proximal sliding displacement.

Specimens were instrumented on the Electro Force 3510 biomechanical testing machine (Bose Corporation, USA) with biomechanical fixtures. This test system had a maximum dynamic load of 7.5 kN, a dynamic displacement of 50 mm, and a test frequency from static to 100 Hz. The vertical loading force line was coincided with the mechanical axis of the femur specimen to better simulate a single‐leg stance model.

#### *Torsional Loading Test*

Five Nm of torque was preloaded for 5 s and up to 20 Nm with internal rotation at a speed of 20 degrees/min. The test was stopped when one of the following occurred: (i) the torque reached 20 Nm; or (ii) the internal fixation failed. Each specimen was subjected to five torsional loading tests and rested for 5 min after each test to eliminate the effect of the last twist on the latter.

#### *Axial Loading Test*

The axial load of 100 N was preloaded and then proceeded at a rate of 10 mm/min. This ceased when the axial load reached 500 N or internal fixation failure occurred. Similarly, each specimen was subjected to five axial loading tests with a 5‐min interval for each test.

#### *Cyclic Axial Loading Test*

Cyclic axial loading was performed after the axial loading test. Each construct was subjected to 300 N up to 1800 N with the loading increment of 100 N, at 1 Hz for 100 cycles, allowing 10‐s rest between each load increment.

After cyclic loading, if the constructs did not fail, they were loaded under axial loading at the rate of 10 mm/min until failure in a single‐leg stance position. If the construct did not fail, the following conditions were defined: the total deformation reached 0.5 cm; the implant bent; or acute change in load‐deformation curve. Then we continued with the next cyclic axial loading.

### *Measurement and Calculation*

We detected the twisted angle and deformation, and calculated the average torsional axial stiffness. The software (Bluehill and MAX) setup of the loading machine, the sample mounting, and the stiffness calculation procedures remained unchanged throughout the tests. We calculated the implant total deformation after the cyclic load. Irreversible deformation was calculated by subtracting the initial deformation from deformation present after the failure of the femur‐implant construct in cyclic loading.

### *Statistical Method*

SPSS 18.0 software (SPSS, USA) was used to perform the data analysis. All statistical analysis was performed using independent‐samples *t*‐test. A value of *P* < 0.05 was considered statistically significant.

## Results

### *Bone Quality Comparison*

**Figure 3:**
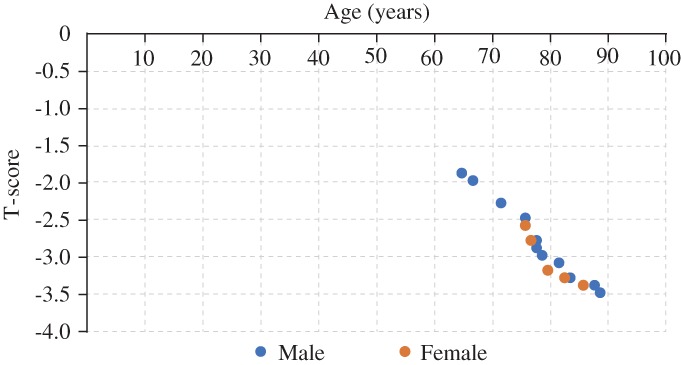
Bone mineral density T‐score distribution of each femoral specimens were relatively concentrated. There was no significant difference in bone density between each specimen.

The results of DEXA examination showed that the bone density T‐score of 16 femoral specimens was 2.86 ± 0.48. The bone mineral density (BMD) distribution of specimens of different ages and genders are shown in the scatter diagram in Fig. 3.

### *Torsional and Axial Loading Tests*

**Figure 4:**
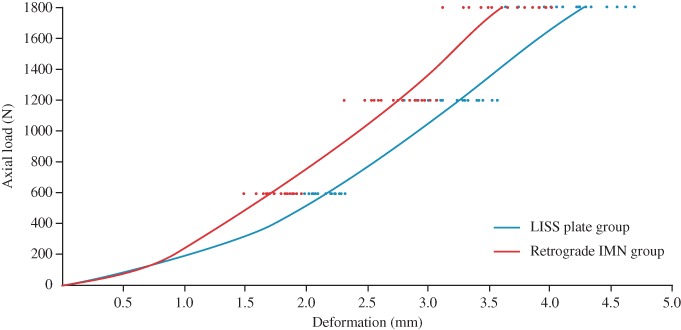
The axial loading‐deformation curves of less invasive stabilization system (LISS) plate group and retrograde IMN group. Through the slope of the curve, the retrograde IMN group can be visually compared with greater axial stiffness.

**Figure 5:**
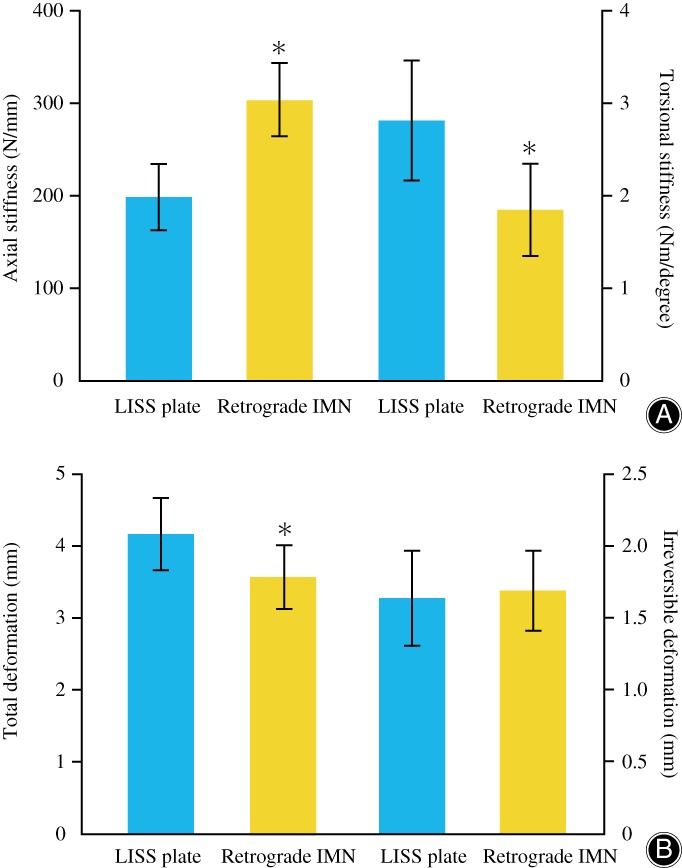
Comparison of stiffness and deformation between less invasive stabilization system (LISS) plate group and retrograde intramedullary nailing (IMN) group. (A) Axial stiffness and torsional stiffness (B) Total deformation and irreversible deformation. *Significant difference.

The biomechanical data on a total of 32 femurs (16 matched pairs) were analyzed. No catastrophic failure was observed in each group after either torsional or axial loading tests. The axial loading‐deformation curves of the LISS plate group and the retrograde IMN group are shown in Fig. 4. We could intuitively find that the slope of the retrograde IMN group was significantly higher than that of the LISS plate group; the axial stiffness of the retrograde IMN was greater. The mean torsional stiffness of the LISS plate group was 34.1% greater than that of retrograde IMN group (2.90 *vs* 1.91 Nm/degree, *P* = 0.002), but the mean axial stiffness was significant greater for the retrograde IMN group (199.16 *vs* 303.93 N/mm, *P* < 0.001). (Table 1, Fig. 5A).

**Table 1:** Stiffness and deformation of LISS plate and retrograde IMN

Stiffness and deformation	LISS plate (*n* = 16)	Retrograde IMN (*n* = 16)
Axial stiffness (N/mm)		
Mean	199.16	303.93
SD	35.55	39.24
Difference	104.78	
*P*‐value*	<0.001	
Torsional stiffness (Nm/degree)		
Mean	2.90	1.91
SD	0.67	0.52
Difference	0.99	
*P*‐value*	0.002	
Total deformation (mm)		
Mean	4.17	3.57
SD	0.51	0.44
Difference	0.60	
*P*‐value*	0.014	
Irreversible deformation (mm)		
Mean	1.64	1.69
SD	0.33	0.28
Difference	0.06	
*P*‐value*	0.699	

* Independent Student *t*‐test

IMN, intramedullary nailing; LISS, less invasive stabilization system; *n*, number of specimens for analysis.

### *Cyclic Axial Loading Test*

**Figure 6:**
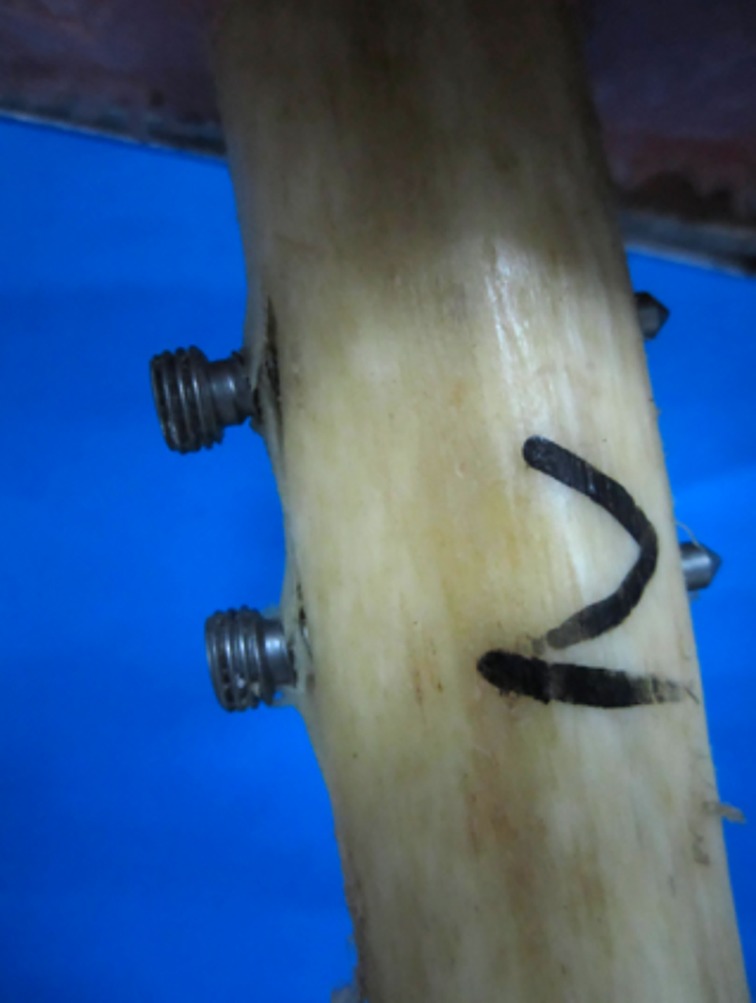
The cortical bone around the proximal screw cap of the retrograde intramedullary nail was cleft under cyclic loading in 1 case.

The total deformation of the LISS plate caused by cyclic axial loading was greater than that of retrograde IMN, and the difference was statistically significant (4.17 *vs* 3.57 mm, *P* = 0.014). Non‐significantly less mean irreversible deformation was detected for LISS plates than for retrograde IMN (1.64 *vs.* 1.69 mm, *P* = 0.699) (Table 1, Fig. 5B). No catastrophic failure was observed in each group after all cyclic axial loading tests. However, 1 specimen of retrograde IMN group had a deformation of more than 0.5 cm at 1700 N of cyclic loading; the cortical bone around the proximal screw cap was cleft (Fig. 6).

### *Failure Load Test*

Failure loads of the constructs were significantly different between the two groups (LISS plate: 2941 ± 128 N; retrograde IMN: 4022 ± 176 N, *P* < 0.001). After the ultimate load, the LISS plate group showed slight curvature at the fracture gap in 1 case and proximal screw loosening in 3 cases; 6 cases in the retrograde IMN group showed cortical fractures around the proximal locking screw. No other specimens showed obvious damage, but the load curve of real‐time observations from the mechanical software demonstrated extreme decline.

## Discussion

### *Comparison of LISS Plate and Retrograde Intramedullary Nail Biomechanical Properties*

Clinical research was performed to compare the efficacy of retrograde IMN (59 cases) and LISS plates (56 cases) in the treatment of distal femoral fractures. The results showed that the techniques were effective for type A fractures, with no significant difference in the implants, and revealed that the outcome largely depended on surgical technique rather than the choice of implant^10^. Many scholars have evaluated the clinical efficacy of LISS plate in the treatment of distal femoral fractures, with clinical healing rates of 85%–100%^14^.

Unger *et al*.^18^ report that bone specimens soaked with formalin, glycerol, and alcohol did not differ in stiffness from fresh bone but were reduced by 20% in terms of elastic energy absorption. In our study, to simulate the real‐life situation, we chose to use anti‐corrosion cadaver bone instead of artificial bone. As a well‐established modeling technique for biomechanical study of the distal femur, a 1‐cm transverse osteotomy was created 6 cm from the intercondylar fossa^14^, ^19^, ^20^, ^21^, ^22^. Generally, this kind of absolute bone defect is rarely encountered in the clinic, and fracture fragments fill the gap, so the stress stiffness of the implants inside the body would be greater than that in our biomechanical study. The advantage of using this model was eliminating the transduction of the bone mass in the fracture gap, so that all the loads were loaded onto the implants during the test, which might reflect the mechanical properties of the two internal fixation techniques to the greatest extent. We applied two representative internal fixation techniques on two femoral specimens of the same cadaver for torsional, axial, and cyclic loading tests to eliminate inter‐group errors as much as possible.

The average torsional stiffness of LISS plates was 34.1% larger than that of retrograde IMN, and the axial stiffness was significant smaller than that of the retrograde IMN. Wähnert *et al*.^23^ used the same fracture model and compared the biomechanical properties of three different intramedullary nails (SCN, DFN, and T2 intramedullary nails) and the AxSOS locking plate, and found that the torsional stiffness of the locking plate was greater than for the intramedullary fixation system; SCN had the largest axial stiffness, followed by DFN, T2 intramedullary nails, and the locking plate, which was consistent with our experimental results. Similar results have been reported in several other biomechanical and clinical studies^14^, ^15^, ^24^, ^25^, ^26^.

The total deformation of the LISS plate was significantly greater than that of retrograde IMN in the cyclic loading test (4.17 *vs*. 3.57 mm, *P* = 0.014), but not statistically significant in irreversible deformation (*P* > 0.05). The LISS plate had greater torque and, thus, produced greater total deformation under the same cyclic load. Because LISS plates had no contact with the surface of femur, they were more flexible, so the difference in irreversible deformation was not significant.

Zlowodzk *et al*.^17^ compared the biomechanical properties of LISS plates and retrograde IMN on fresh frozen cadavers. They also simulated an AO/OTA33‐A3 femoral supracondylar fracture model of 1‐cm defect. The results showed that the total deformation of the LISS plate was larger than that of retrograde IMN, and the irreversible deformation was not significant, which was consistent with our results. At the same time, they found that retrograde IMN was more prone to failure than LISS plates, and speculated a trade‐off between axial stiffness and failure load of the internal implant. Consistent results were also obtained in this study; only 1 construct in the retrograde IMN group showed cortical bone cleft around the proximal screw head.

### *Stability Comparison of LISS Plate and Retrograde Intramedullary Nail*

The deformation of retrograde IMN under the same axial load was less than that of the LISS plate due to the small torque, resulting in a significant difference in axial stiffness. In our tests, the torsional stiffness was significantly larger in the LISS plates which showed more stability under shear force. However, Taylor *et al*.^27^ found through clinical trials that the biomechanical properties of torsional stiffness did not appear to be so important during postoperative recovery; they also found that the maximum value of the torsional load during one leg stance did not exceed 10 Nm. Referring to the Wikens *et al*.^19^ test protocol for the biomechanical parameters of the distal femur, we applied a torsional load of 20 Nm, resulting in no single catastrophic failure. Therefore, we believed that LISS plates and retrograde IMN were stable under physiological torsional loading.

In this study, we simplified the force of internal fixation *in vivo* without simulating any muscle loads during the above testing procedures. At the same time, we only tested the compressive stress and torsional shearing force of the simulated single‐leg stance (fully extended position of the knee joint). Other complex movements, such as the force in the process of going up and down stairs, were not tested, because in the early postoperative period, patients rarely carried out such complex movements.

This study did not consider the effect of the *in vivo* environment on the biomechanical properties of the internal fixation during the healing period. Although balance between the strength of fixation and micro‐motion at the fracture end has not been found, excessive dislocation of fracture ends can obviously lead to nonunion, malunion or even implant failure^16^, ^28^. In addition, excessive micro‐motion was beyond the scope of tissue compensation, causing apoptosis and ultimately leading to fracture nonunion or implants failure for revision surgery^29^, ^30^. Fracture site, fracture severity, and bone quality were all factors in choosing the internal fixation method in clinical treatment. Ahmadi *et al*.^31^ pointed out that retrograde IMN was more suitable for fractures with tumor‐type defects, while the LISS plate was more suitable when the distance of the fracture end was far away or with less bone tissue at the distal end.

### *Comparison of Advantages and Disadvantages of Two Fixations*

Kregor *et al*.^32^ treated 103 cases of distal femoral fractures with LISS plates. The LISS plates used locking nail technology, which did not compress the bone under the plate. The distal seven angled locking screw holes made it more suitable for the treatment of osteoporotic fractures, and extensive incision was not required for distal femoral fractures. The internal fixation required only percutaneous implantation, which minimized the damage to the fracture segment and protected the local blood supply to reduce the infection rate.

The reasons for the loosening of the proximal locking screws of the LISS plate in 3 cases may include incorrect placement of the LISS plate, resulting in incomplete attachment of bone, or when the self‐tapping screws were used, the local temperature being too high, causing the cortical bone of the nail to be damaged, so that the local holding force was reduced, resulting in loosening of the nail path^33^.

Intramedullary fixation could protect the blood supply around the fracture to the greatest extent, and, at the same time, achieved multi‐angle interlocking. The nail insertion hole was located in the intercondylar fossa, which did not involve the articular cartilage, minimizing the adhesion of soft tissue around the joint and facilitating early functional mobilization. However, Helfet *et al*.^34^ point out that retrograde IMN should not be used for type B fractures of the distal femur, complicated comminuted fractures of type C3, and low transcondylar fractures, because the retrograde nail device could further damage the articular surface and the stability of the reconstruction.

The results for axial failure load in this study further indicated that intramedullary fixation was significantly superior to extramedullary fixation. This might be due to the fixation with IMN, which was closer to the force line of the lower limb, providing strong fixation; static fixation was also beneficial to fracture healing^35^. At the same time, according to the ultimate load result, it could assist orthopaedic surgeons in the choice of weight‐bearing timing and mode after operation.

### *Limitations*

There are some limitations in our study as follows. No fresh cadaveric bone was used. All soft tissues and fracture fragments were removed for simulating fracture models and biomechanical testing, which is different from the real clinical situation. We only tested these models in neutral position; other positions such as flexion, abduction, and adduction were not included.

In fixing comminuted femoral supracondylar fractures, both LISS plates and retrograde IMN could provide sufficient biomechanical stability, but the retrograde IMN construct was superior to the LISS plate construct because of the smaller total deformation and the larger irreversible deformation, and the retrograde IMN construct could bear more failure load.
